# Supplementary material for: Are degree of urbanisation and travel times to healthcare services associated with the processes of care and outcomes of heart failure? A retrospective cohort study based on administrative data
Source: PLoS One. 2019 Oct 28;14(10):e0223845. doi: 10.1371/journal.pone.0223845 (PMC6816546; doi:10.1371/journal.pone.0223845)

Figure A. Average elevation for the 45 municipalities of the Local Healthcare Authority of Bologna: plain (<80 m), hills (80-440 m) and mountains (441-1000 m).

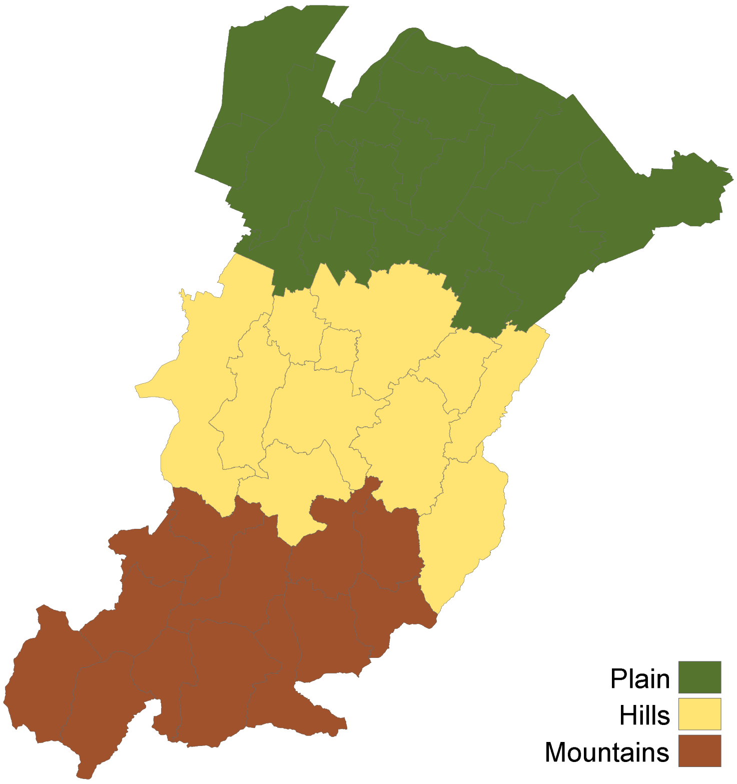

Figure B. Degree of urbanisation for the 45 municipalities of the Local Healthcare Authority of Bologna: densely populated (cities), intermediate density (towns and suburbs) and sparsely populated (rural areas).

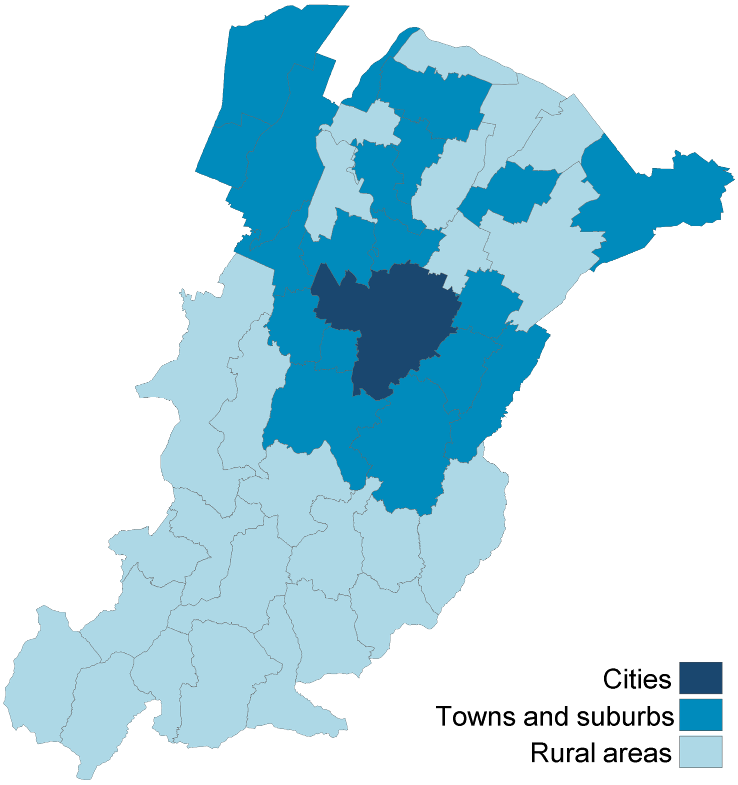

Supplement: S1 Fig — The shapefiles used to make this figure are made publicly available by the Italian National Institute of Statistics. Administrative boundaries reproduced from [31] under a CC BY license, with permission from the Italian National Institute of Statistics, original copyright 2019. (PDF) [file pone.0223845.s002.pdf]
