# Supplementary material for: Are degree of urbanisation and travel times to healthcare services associated with the processes of care and outcomes of heart failure? A retrospective cohort study based on administrative data
Source: PLoS One. 2019 Oct 28;14(10):e0223845. doi: 10.1371/journal.pone.0223845 (PMC6816546; doi:10.1371/journal.pone.0223845)
Supplement: S2 Fig — GP, general practitioner. (PDF) [file pone.0223845.s003.pdf]

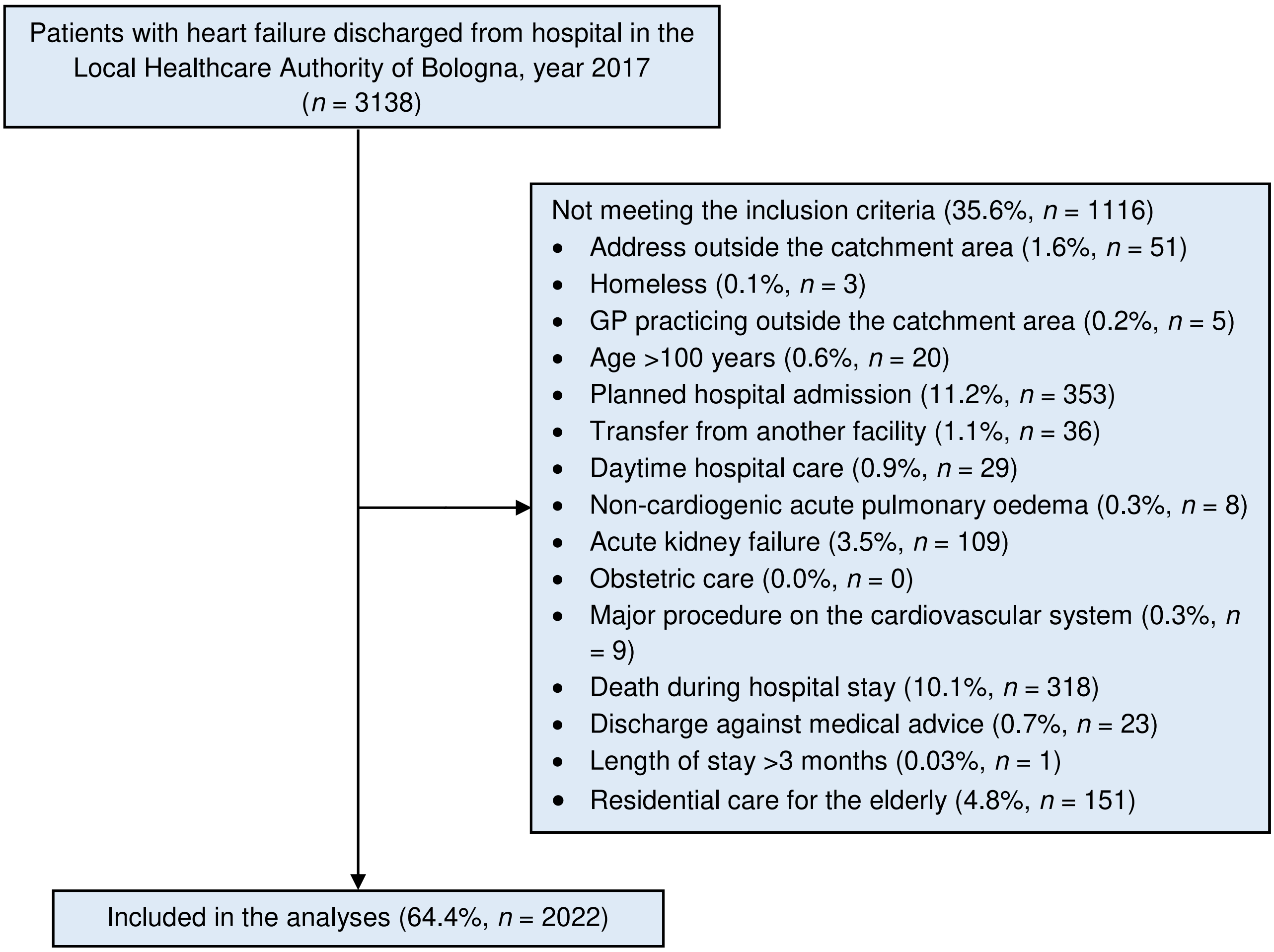
Patients with heart failure discharged from hospital in the Local Healthcare Authority of Bologna, year 2017 (n = 3138)
Not meeting the inclusion criteria (35.6%, n = 1116)
Address outside the catchment area (1.6%, n = 51)
Homeless (0.1%, n = 3)
GP practicing outside the catchment area (0.2%, n = 5)
Age >100 years (0.6%, n = 20)
Planned hospital admission (11.2%, n = 353)
Transfer from another facility (1.1%, n = 36)
Daytime hospital care (0.9%, n = 29)
Non-cardiogenic acute pulmonary oedema (0.3%, n = 8)
Acute kidney failure (3.5%, n = 109)
Obstetric care (0.0%, n = 0)
Major procedure on the cardiovascular system (0.3%, n = 9)
Death during hospital stay (10.1%, n = 318)
Discharge against medical advice (0.7%, n = 23)
Length of stay >3 months (0.03%, n = 1)
Residential care for the elderly (4.8%, n = 151)
Included in the analyses (64.4%, n = 2022)
