# Supplementary material for: Are degree of urbanisation and travel times to healthcare services associated with the processes of care and outcomes of heart failure? A retrospective cohort study based on administrative data
Source: PLoS One. 2019 Oct 28;14(10):e0223845. doi: 10.1371/journal.pone.0223845 (PMC6816546; doi:10.1371/journal.pone.0223845)
Supplement: S1 Table — (PDF) [file pone.0223845.s004.pdf]

Database	Description
Hospital discharges	Information for each inpatient discharged from either public or accredited private hospitals. Diagnoses and procedures are classified in the ICD-9-CM. Since 1995 the Diagnosis Related Group system has been systematically used to allocate funds to hospitals and to monitor quality of care and outcomes.
Outpatient care	Individual data on services provided to non-admitted, non-emergency patients registered for care in outpatient care settings or hospitals.
Emergency room	Individual data on emergency room visits not resulting in inpatient admission.
Vital registration	Information on patients' date, place and cause of death classified in the ICD-10.
Outpatient prescription drugs	Individual data on prescriptions (substance name, ATC System code—version 2013, trade name, date of prescription filling, and number of packages) and prescribers. Only drugs reimbursed by the healthcare system that are prescribed by primary care physicians or specialists, or directly delivered by hospital pharmacies.
