# Supplementary material for: Are degree of urbanisation and travel times to healthcare services associated with the processes of care and outcomes of heart failure? A retrospective cohort study based on administrative data
Source: PLoS One. 2019 Oct 28;14(10):e0223845. doi: 10.1371/journal.pone.0223845 (PMC6816546; doi:10.1371/journal.pone.0223845)
Supplement: S2 Table — ER, emergency room; GP, general practitioner; FUP, follow-up. (PDF) [file pone.0223845.s005.pdf]

Outcome	Urbanisation and travel times	Other predictors
• Cardiology visit	• Urbanisation (urban, peri-urban, rural)<br>• Nearest cardiology ward or outpatient cardiology service	
• Hospital readmission<br>• ER visit<br>• Mortality	• Urbanisation (urban, peri-urban, rural)<br>• Nearest ER<br>• Patient's GP nearest practice<br>• Nearest outpatient cardiology service or ambulatory care nursing practice	• Cardiology visit during FUP
