# Supplementary material for: Are degree of urbanisation and travel times to healthcare services associated with the processes of care and outcomes of heart failure? A retrospective cohort study based on administrative data
Source: PLoS One. 2019 Oct 28;14(10):e0223845. doi: 10.1371/journal.pone.0223845 (PMC6816546; doi:10.1371/journal.pone.0223845)
Supplement: S3 Table — (PDF) [file pone.0223845.s006.pdf]

Table A. Comorbidities retrieved from hospital discharge records: 31 Elixhauser conditions plus acute myocardial infarction, cerebrovascular diseases, dementia, and leukaemia (Quan et al. 2005, for ICD-9-CM codes).

Clinical condition
History of congestive heart failure
Cardiac arrhythmias
Valvular disease
Pulmonary circulation disorders
Peripheral vascular disorders
Hypertension, uncomplicated
Hypertension, complicated
Paralysis
Other neurological disorders
Chronic pulmonary disease
Diabetes, uncomplicated
Diabetes, complicated
Hypothyroidism
Chronic kidney disease
Liver disease
Peptic ulcer disease excluding bleeding
AIDS/HIV
Lymphoma
Metastatic cancer
Solid tumour without metastasis
Rheumatoid arthritis/collagen vascular diseases
Coagulopathy
Obesity
Weight loss
Fluid and electrolyte disorders
Blood loss anaemia
Deficiency anaemia
Alcohol abuse
Drug abuse
Psychoses
Depression
Acute myocardial infarction
Cerebrovascular diseases
Dementia
Leukaemia

Table B. ATC codes for identification of medication use before heart failure.

Medication	ATC codes
Antidiabetic drugs	A10
Drugs for cardiac therapy	C01
Drugs for obstructive airway diseases	R03
Diuretics	C03
β-blockers	C07
Angiotensin-converting enzyme inhibitors/angiotensin receptor blockers	C09
Calcium channel blockers and other antihypertensive drugs	C02, C08
Statins	C10AA
Antiplatelet drugs	B01AC
Oral anticoagulants	B01AA03, B01AA07, B01AE
