# Supplementary material for: Are degree of urbanisation and travel times to healthcare services associated with the processes of care and outcomes of heart failure? A retrospective cohort study based on administrative data
Source: PLoS One. 2019 Oct 28;14(10):e0223845. doi: 10.1371/journal.pone.0223845 (PMC6816546; doi:10.1371/journal.pone.0223845)
Supplement: S4 Table — (PDF) [file pone.0223845.s007.pdf]

Table A. Distribution of patient's comorbidities.

**Comorbidities**	***n***	%
Cardiac arrhythmias	1141	56.4
Hypertension, either complicated or uncomplicated	1010	50.0
Chronic pulmonary disease*	710	35.1
History of congestive heart failure	663	32.8
Diabetes, either complicated or uncomplicated*	606	30.0
Chronic kidney disease	554	27.4
Valvular disease	528	26.1
Cerebrovascular disease	260	12.9
Acute myocardial infarction	239	11.8
Dementia	201	9.9
Peripheral vascular disease	153	7.6
Deficiency anaemia	146	7.2
Metastatic and non-metastatic tumours, incl. lymphoma and leukaemia	141	7.0
Fluid and electrolyte disorders	119	5.9
Hypothyroidism	102	5.0
Blood loss anaemia	97	4.8
Pulmonary circulation disorders	83	4.1
Neurological disorders, incl. paralysis	76	3.8
Obesity	75	3.7
Depression	65	3.2
Liver disease	55	2.7
Rheumatoid arthritis/collagen vascular diseases	29	1.4
Coagulopathy	26	1.3
Weight loss	18	0.9
Drug/alcohol abuse	12	0.6
Psychoses	12	0.6
Peptic ulcer disease, excl. bleeding	0	0.0
AIDS/HIV	0	0.0

* In addition to hospital discharge records, filled prescriptions were used to track diabetes (antidiabetic drugs) and chronic pulmonary disease (drugs for obstructive airway diseases), two conditions that are managed to a large extent by general practitioners.

Table B. Distribution of patient's previous drug therapies.

**Drug classes**	***n***	%
Diuretics	1280	63.3
β-blockers	1204	59.5
Angiotensin-converting enzyme inhibitors/angiotensin receptor blockers	1069	52.9
Antiplatelet drugs	784	38.8
Statins	646	31.9
Calcium channel blockers	563	27.8
Oral anticoagulants	516	25.5
Antidiabetic drugs	477	23.6
Drugs for obstructive airway diseases	454	22.5
Drugs for cardiac therapy	412	20.4
