# Supplementary material for: Are degree of urbanisation and travel times to healthcare services associated with the processes of care and outcomes of heart failure? A retrospective cohort study based on administrative data
Source: PLoS One. 2019 Oct 28;14(10):e0223845. doi: 10.1371/journal.pone.0223845 (PMC6816546; doi:10.1371/journal.pone.0223845)
Supplement: S5 Table — (PDF) [file pone.0223845.s008.pdf]

Table A. Relationship of processes of care, urbanisation levels and travel times with cardiology visit within 6 months of heart failure discharge.

Processes of care, urbanisation and travel times	Cardiology follow-up visit	
	OR*	99% CI
Discharge from cardiology	1.69†	1.26-2.29
Inpatient intensive care	0.60	0.34-1.07
HF care pathway		
No	1.00	
Clinic-based	1.69†	1.02-2.80
Home-based	1.09	0.59-2.02
General home care		
No	1.00	
Occasional	0.68	0.33-1.40
Full-time	0.92	0.71-1.19
Degree of urbanisation		
City	1.00	
Towns or suburbs	1.17	0.90-1.52
Rural area	1.42‡	1.03-1.96
Travel time to nearest cardiology service, either inpatient or outpatient		
Very short (≤5 min)	1.00	
Short (>5-10 min)	0.86	0.67-1.10
Medium (>10-20 min)	0.95	0.68-1.34
Long (>20-30 min)	0.63	0.35-1.14
Very long (>30 min)	0.54	0.17-1.67

* Adjusted for age, sex, length of stay, and dementia.
† Significant at the 0.01 level.
OR, odds ratio; *CI*, confidence interval; *HF*, heart failure.

Table B. Relationship of processes of care, urbanisation levels and travel times with hospital readmissions, emergency room visits and mortality within 6 months of heart failure discharge.

Processes of care, urbanisation and travel times	All-cause unplanned readmission		ER visit		All-cause mortality	
	OR*	99% CI	OR†	99% CI	OR‡	99% CI
Discharge from cardiology	0.77	0.53-1.12	0.68	0.44-1.06	0.89	0.50-1.57
Inpatient intensive care	1.13	0.59-2.15	1.58	0.78-3.19	0.92	0.31-2.72
Cardiology visit during FUP	0.86	0.63-1.17	0.99	0.70-1.40	0.53§	0.32-0.87
Use of ACEIs/ARBs during FUP	0.94	0.74-1.20	0.77	0.58-1.01	0.65§	0.45-0.93
Use of β-blockers during FUP	0.93	0.75-1.15	0.93	0.72-1.20	0.75	0.56-1.01
HF care pathway						
No	1.00		1.00		1.00	
Clinic-based	0.77	0.41-1.43	1.11	0.56-2.20	1.05	0.30-3.65
Home-based	0.75	0.41-1.37	1.06	0.53-2.09	1.25	0.67-2.34
General home care						
No	1.00		1.00		1.00	
Occasional	1.43	0.81-2.55	0.87	0.37-2.03	1.52	0.72-3.24
Full-time	1.44§	1.14-1.81	1.26	0.95-1.67	2.11§	1.51-2.94
Degree of urbanisation						
City	1.00		1.00		1.00	
Towns or suburbs	0.77	0.57-1.04	1.10	0.37-2.03	0.96	0.63-1.48
Rural area	0.85	0.60-1.19	1.26	0.95-1.67	0.96	0.57-1.53
Travel time to nearest ER						
Very short (≤5 min)	1.00		1.00		1.00	
Short (>5-10 min)	0.96	0.66-1.39	0.76	0.48-1.20	0.83	0.50-1.39
Medium (>10-20 min)	1.13	0.77-1.67	0.87	0.55-1.37	1.13	0.66-1.93
Long (>20-30 min)	1.36	0.79-2.32	0.92	0.49-1.74	2.08	0.99-4.35
Very long (>30 min)	1.03	0.45-2.37	0.22	0.04-1.11	0.66	0.15-2.94
Travel time to nearest practice of the patient's GP						
Very short (≤5 min)	1.00		1.00		1.00	
Short (>5-10 min)	1.04	0.79-1.36	1.10	0.80-1.52	1.18	0.81-1.71
Medium (>10-20 min)	1.17	0.83-1.65	0.92	0.60-1.42	1.14	0.70-1.83
Long (>20-30 min)	1.59	0.70-3.61	0.65	0.21-1.99	0.72	0.20-2.58
Very long (>30 min)	1.22	0.42-3.54	0.37	0.06-2.46	3.42	0.87-13.5
Travel time to nearest outpatient service, either cardiologist or non-cardiologist						
Very short (≤5 min)	1.00		1.00		1.00	
Short (>5-10 min)	0.87	0.69-1.10	0.98	0.74-1.30	1.12	0.81-1.57
Medium (>10-20 min)	0.83	0.54-1.28	0.96	0.59-1.55	0.60	0.31-1.14
Long (>20-30 min)	1.10	0.49-2.49	0.87	0.25-3.01	1.61	0.51-5.03

* Adjusted for age, sex, length of stay, history of heart failure, diabetes, chronic kidney disease, and previous use of ACEIs/ARBs.

† Adjusted for age, sex, and length of stay.

‡ Adjusted for age, sex, length of stay, history of heart failure, cardiac arrhythmias, chronic kidney disease, dementia, and previous use of diuretics/statins.

§ Significant at the 0.01 level.

FUP, follow-up; *ACEIs/ARBs*, angiotensin-converting enzyme inhibitors/angiotensin receptor blockers; *ER*, emergency room; *GP*, general practitioner.
