# Supplementary material for: Are degree of urbanisation and travel times to healthcare services associated with the processes of care and outcomes of heart failure? A retrospective cohort study based on administrative data
Source: PLoS One. 2019 Oct 28;14(10):e0223845. doi: 10.1371/journal.pone.0223845 (PMC6816546; doi:10.1371/journal.pone.0223845)
Supplement: Attachment — Submitted filename: Reply 2019.09.09.doc [file pone.0223845.s009.doc]

**ACADEMIC EDITOR**

**We thank the Editor for the helpful comments.**

1) It is acceptable that not all information on the methods is provided in the text if previously published. Still, the readers must be able to understand the methods without reading the cited paper. This means that that a short description of the according parts of the methods is required. Only citing a previous study is not sufficient.

**We thank the Editor for his suggestion. We have now provided a more accurate description of the statistical methods used for confounder selection (Ln 229-34).**

2) The authors include some limitations of their analysis but lack to mention all of them. The reviewers addressed some of them and they also made suggestions that can possibly not be addressed based on the administrative data set. The authors must clearly address this. In this regard, a very important shortcoming is that there is no information on the severity of HF.

**We have now mentioned among the study limitations the absence of information on HF severity and other relevant information, including GP visits and presence of caregivers (Ln 334-7, 350-1, 381-5).**

3) Instead of repeating findings previously mentioned as part of the discussion / conclusion, the authors should focus more on the reasons why they think that their findings are in some contrast to other findings and possibly also their expectations (a clear hypothesis would help in this regard), more thoughts about the potential clinical implications and future perspectives, considering expected changes in healthcare in the future (as in part also addressed by the reviewers). If the authors do not want to extend too much regarding length of the text, the introduction can be shortened. This particularly refers to the extensive discussion about the burden of heart failure. This is well known, mentioned in thousands of papers, and can therefore be shortened significantly.

**We thank the Editor for his thoughtful suggestion. We have enhanced the conclusions to highlight the practical implications of our findings, as well as the future perspective related to health resource decline and population ageing (Ln 404-8). In particular, we argued that healthcare delivery could be optimised by prioritising quality, not only quantity of services being provided. The contrast with other findings is addressed in Ln 364-9, 374-7 and 396-8, where we discuss SES distribution, universal health coverage, multidisciplinary interventions and relatively short driving distances. Lastly, as suggested, the introduction has been shortened (Ln 49-52).**

4) Please check that the references to tables and figures are correct. Suppl. text 1 is a figure. Please check that the manuscript follows the requirements of the journal regarding format, placement of tables, figures, references etc

**Supplementary files have been renamed according to their content. We have also double-checked all formatting guidelines, including references and figure requirements.**

**REVIEWER #1**

Many thanks for the opportunity to review this interesting manuscript examining the relationship between urbanisation and travel time with processes of care and heart failure outcomes. In general it was well written and structured with transparency and rigour from data to conclusion. I would like to make few comments which I would like your consideration.

**We thank the reviewer for this positive comment. We hope that we have addressed adequately each of the issues she/he raised.**

In terms of participant characteristics, it would have been beneficial to know NYHA class &/or the EF. This was particularly pertinent as on review of Table 1, 18 (4.3%) of rural compared to 27 (2.8) city and 22 (3.4) towns & suburbs were discharged from intensive care. Was there a difference in the "sickness" of the patients"?

**Unfortunately, information on NYHA class and ejection fraction is not routinely collected in the Italian hospital discharge records. We have now mentioned this major shortcoming in the discussion (Ln 334-7).**

Figure 1 demonstrated a numerous GP practices and centralised cardiology wards. Can I ask why GP visits were not recorded or included as a study outcome?

**We thank the reviewer for raising this point. In Italy, most GPs use dedicated computer programmes to manage their rosters, appointments and clinical data. However, these programmes cannot be accessed by the Local Healthcare Authorities. The lack of information on GP visits has been now addressed in the discussion (Ln 381-5).**

Use of Doppler echocardiogram during hospital stay noted a potential confounder- why? Surely all HF patients will have an objective assessment.

**We thank the reviewer for her/his remark. Initially we included Doppler echocardiography in the analysis, but then decided to discard it because most patients with heart failure get this evaluation, as the reviewer correctly pointed out. Unfortunately, we forgot to erase this information from our previous versions of the manuscript. The text has been amended accordingly (Ln 178-9 and table footnotes).**

Results were displayed appropriately in table with main point summarised in text. Please amend Table 4 as "degree of urbanisaton" data is repeated. I am unsure of the value of SI table. Also please refer to S1 figure in main text to ensure reader knows to access the information. It is important to ensure clear land marking of the supplementary material.

**We thank the reviewer for noticing these inaccuracies—Table 4 has been corrected, and supplementary files have been renamed according to their content. We think that S1 Table might be of some value for those who are not familiar with multivariate statistics, that is, the simultaneous analysis of multiple outcomes. Because making ourselves clear is our priority, we would rather keep this synthesis matrix as supporting information.**

Results inform conclusions that travel time and urbanisation had no effect on processes of care and outcomes. Cardiology visits were more frequent among rural patients. It would have been interesting to hear your thoughts on how this will change in the future in light of declining health resources and a growing elderly population with multiple comorbidities.

**We thank the reviewer for her/his suggestion. We have enhanced the conclusions to highlight the practical implications of our findings, as well as the future perspective related to health resource decline and population ageing (Ln 404-8). In particular, we argued that healthcare delivery could be optimised by prioritising quality, not only quantity of services being provided.**

**REVIEWER #2**

General remark: authors assume that readers have knowledge how local health care is organised. Despite they tried to optimally explain the organisation, it is not always evident to understand the organisation. Some questions reflect on this topic.

**We thank the reviewer for the helpful comments. We hope that we have better clarified how healthcare services are organised in our Local Healthcare Authority.**

Sentence 63-65: Would the authors please rephrase: 'However, these studies focused mainly on patients with cancer and were carried out using very different data sources and methods, therefore more research is needed to obtain further evidence on this topic.'

**The sentence has been rephrased as follows: “However, this review included mainly cancer research studies relying on different data sources and variables” (Ln 58-9).**

Sentences 71-72: 'Also, there is a lack of evidence regarding transport accessibility measured as the actual distance between the patient's home and the healthcare facility.'

**The sentence has been rephrased as follows: “A potential limitation of these studies is the lack of information regarding transport accessibility measured as the actual distance between the patient’s home and the healthcare facility” (Ln 64-6).**

Sentences 177-180: Potential confounders: In order to minimise the potential confounding of individual characteristics on the association of urbanisation level and travel times with outcomes, we retrieved some patient baseline characteristics. These included:

1. Demographic characteristics (age, sex and citizenship)

**The sentence has been rephrased as follows: “We collected some patient baseline characteristics to reduce the potential source of confounding. These included: 1) Age; 2) Sex; 3) Citizenship” (Ln 172-6).**

Sentences 388-343: In the discussion authors discussed the travel burden for elderly patients, and the fact that elderly patients often must be accompanied by their caregivers...

Reviewer: Accessibility of transport also means the easiness of getting transport. In case of elderly patients the presence of informal caregivers might be of utmost importance. The authors included 'citizenship' into the confounders. Yet, citizenship does not cover the presence / availability of informal caregivers to transport patients to a health care facility... Authors discuss the importance of family members / informal caregivers in relation to the transport.

I would like to ask the authors to add ‘presence of informal caregivers’ to the confounders and investigate / show the results of this important aspect.

**We thank the reviewer for raising this important point. Unfortunately, the presence of informal caregivers is not reported in the Italian hospital discharge records. To the best of our knowledge, administrative data sources of many other countries also lack this information. We have now mentioned this major shortcoming in the discussion (Ln 350-1).**

Do the authors have information about the ratio of no-shows in the several urbanisation degrees. Do patients of the city less frequently have a no-show visit compared to rural patients? If patients do not visit health care facilities (due to transport issues, for example due to lacking transport support), this might be considered as a bad result, which now is not visible in the results.

**Unfortunately, this information is not available because our administrative databases include only visits that are booked AND provided to the patient. In Emilia-Romagna there is a regional law that should discourage no-shows, because patients that do not show up without cancellation have to pay the full patient contribution (Legge regionale 2/2016: “Norme regionali in materia di organizzazione degli esercizi farmaceutici e di prenotazioni di prestazioni specialistiche ambulatoriali” art. 23 comma 3). For this reason, it is reasonable to assume that the number of no-shows is limited in the catchment area of Bologna.**

Table 4 contains double information about 'degree of urbanization': please remove double information.

**We thank the reviewer for noticing the inaccuracy—Table 4 has been corrected.**

Sentence 349-355: Authors discuss about HF-CP and the fact that it is easier to organize HF-CP in rural area. Authors show the health care services in a map: S1 Text. Maps of the Local Healthcare Authority of Bologna, Northern Italy. For me as a reader it is not clear where HF-CP are located. The map shows several services, yet no HF-CP. Please add where HF-CP are organised.

**The HF-CP is a structured multidisciplinary care plan that promotes integration between primary and secondary care, and details essential steps in the care of patients. The GP remains the gatekeeper for the patients and coordinates with cardiologists and nurses for an easier access to consultation and counselling to improve lifestyle and optimise medication adherence. There are no specific HF-CP centres or facilities in the catchment area, because registered patients access GP practices, cardiology services or ambulatory care nursing practices in case they need medical care or counselling. All these aspects have been clarified in the Methods section (Ln 194-203).**

Rural patients are receiving more follow-up visits from a cardiologist. Are cardiologists always functioning into outpatient cardiology services or do they have also consulting hours in other services? Or involved into the HF-CP? Please add this information.

**We have now stated in the Methods section that cardiology follow-up visits can be provided either in inpatient cardiology services or in outpatient cardiology services (Ln 125). Cardiologists in either settings of care can see all patients with HF.**

Authors investigated a very local region. Do the authors have advice for other regions?

**We thank the reviewer for her/his question. Our findings cannot be generalised to isolated and remote communities, but we gave some advice for other regions where driving distances to healthcare facilities are relatively short. In particular, we argued that healthcare delivery could be optimised by prioritising quality, not only quantity of services being provided (Ln 404-8).**
