# Supplementary material for: Are degree of urbanisation and travel times to healthcare services associated with the processes of care and outcomes of heart failure? A retrospective cohort study based on administrative data
Source: PLoS One. 2019 Oct 28;14(10):e0223845. doi: 10.1371/journal.pone.0223845 (PMC6816546; doi:10.1371/journal.pone.0223845)
Supplement: Attachment — Submitted filename: Reply 2019.09.25.doc [file pone.0223845.s010.doc]

**REVIEWER #1**

**We thank the reviewer for the helpful comments.**

Sentence 65: Please remove repetition of the words "relying on different"

**We thank the reviewer for noticing this inaccuracy. The text has been amended.**

Sentence 77: Change the word "founded" to "established"

**The verb has been changed as suggested.**

Sentence 83: Remove the word "However" as not required

**We agree with the reviewer that the two paragraphs are congruent and no adverb is required to link them. The text has been amended.**

Sentence 86: Ensure consistency - consider rephrasing to "The objectives of this study were to investigate whether urbanisation levels and travel time to healthcare services in the LHA of Bologna, are associated with processes of care and outcomes of patients with heart failure".

**We thank the reviewer for raising this point. Our introduction is “travel time-oriented”, that is, we highlight that there is lack of evidence regarding transport accessibility measured as the actual distance between the patient’s home and the healthcare facility. We organised our study aim on purpose to give priority to travel times, and added that urbanisation was also assessed to be consistent with existing literature. We really appreciate the reviewer’s suggestion, but we feel that putting urbanisation level and travel times on the same level in the last paragraph would make the whole introduction section less flowing.**

Sentence 100: Could you provide details of the data sources, perhaps in a table format?

**A new supplementary table (S1) has been included, as suggested.**

Sentence 180: Insert the word "variable" after confounding

**In this sentence, “confounding” is a noun. A number of occurrences where “confounding” is used as a noun can be borrowed from the literature (e.g., *Occup Environ Med* 2003;60:227-34).**

Sentence 212: Please rephrase

**We have rephrased the sentence as follows: “There are no facilities specifically dedicated to the HF-CP: registered patients can access GP practices, cardiology services or ambulatory care nursing practices in case they need medical care or counselling.”**

Sentence 257: Consider using the term pseudonymised instead of "de-identified"

**We thank the reviewer for her or his suggestion. The verb has been changed as suggested.**

Sentence 330: Clarify there are indeed 2 tables "A" and "B" within S4

**This aspect has been clarified for S3, S4 and S5 Tables.**

There are a number of limitations noted within the discussion section. It might be more appropriate to group these together into one paragraph, titled limitations.

**We agree with the reviewer that grouping together all limitations in a single paragraph might be desirable. The journal gives a degree of freedom to the authors, who are not forced to fill prearranged sections when writing the papers. We took advantage of this to write a discussion where methodological pitfalls are in the focus. The reviewer can see that a number of important limitations, such as the lack of relevant clinical information, are at the very beginning of the discussion—the editor asked us to give much relevance to such limitations. For this reason, we have kept the discussion unchanged, but added a subsection where all limitations are now summarised.**

**REVIEWER #2**

I've found one sentence which has to be corrected: Sentence 58-59: double text: “However, this review included mainly cancer research studies relying on different relying on different data sources and variables.”

**We thank the reviewer for noticing this inaccuracy. The text has been amended.**
